# A new species of *Pseudodiaptomus* (Crustacea, Copepoda, Calanoida, Pseudodiaptomidae) from the Prasae River Estuary, Gulf of Thailand

**DOI:** 10.3897/zookeys.338.5531

**Published:** 2013-10-02

**Authors:** Khwanruan Srinui, Shuhei Nishida, Susumu Ohtsuka

**Affiliations:** 1 Institute of Marine Science, Burapha University, Muang, Chonburi 20131,Thailand; 2 Atmosphere and Ocean Research Institute, The University of Tokyo, 5-1-5 Kashiwanoha, Kashiwa 277-8564, Japan; 3 Takehara Marine Science Station, Setouchi Field Science Center, Graduate School of Biosphere Science, Hiroshima University, 5-8-1 Minato-machi, Takehara 725-0024, Japan

**Keywords:** Copepoda, Calanoida, Gulf of Thailand, Prasae River, *Pseudodiaptomus*, new species

## Abstract

A new species of the calanoid copepod genus *Pseudodiaptomus* was collected from the Prasae River Estuary, Rayong Province, on the eastern coast of the Gulf of Thailand. This species is definitely assigned to the *lobus* species group sensu Walter (1986a). The female of the new species differs from other congeners in the elongate genital double-somite with a blunt process ventrally and the second urosomite about 2.54 times as long as wide. The male is also easily distinguished from other congeners by the structure of the right fifth leg.

The present new species is a euryhaline species and occurred in brackish waters with salinity ranging from 0.7 to 23.3. Its breeding season may be from June to October, as indicated by the presence of egg-sacs.

## Introduction

We have been intensively investigating the taxonomy, biology and ecology of gelatinous and crustacean zooplankters in Thailand since 1997 (Pinkaew et al. 1997, 2000; Pinkaew 2003; Ohtsuka et al. 1999, 2003, 2010, 2012; Fukuoka and Pinkaew 2003; Fukuoka et al. 2005; Nishida and Nishikawa 2011; Nishikawa et al. unpublished). Special attention has been paid to copepods, mysids and rhizostome jellyfish, due to their numerical importance in the plankton communities in the coastal and estuarine waters.

During our survey in estuaries of Thailand in 2004–2012 a new species of the calanoid copepod genus *Pseudodiaptomus* was found at the mouth of Prasae River, Gulf of Thailand. *Pseudodiaptomus* is broadly distributed in freshwater to marine habitats in the Atlantic and Indo-Pacific regions, and frequently comprises a main component in the zooplankton communities (Walter 1987). Recently some pseudodiaptomids have been introduced into new habitats via ballast water: the Indo-West Pacific species *Pseudodiaptomus trihamatus* was found along the Northeastern Coast of Brazil (Medeiros et al. 1991; Medeiros et al. 2006); the West Pacific species *Pseudodiaptomus marinus* has so far been recorded from Hawaii (Jones 1966), San Francisco Bay (Orsi and Walter 1991), Iraq (Khalaf 1992), the southern bight of the North Sea, France (Brylinski et al. 2012), Todos Santos Bay, Baja California (Jiménez-Pérez and Castro-Longoria 2006), and the North Adriatic Sea (Olazabal and Tirelli 2011). In addition the Asian species *Pseudodiaptomus inopinus* has been introducedto Oregon, Washington, and British Columbia Estuaries (Cordell and Morrison 1996); andanother Asian species *Pseudodiaptomus forbesi* has been introduced to the new world, and been devastating the native ecosystems as an invasive alien (Orsi and Walter 1991, Cordell et al. 1992, 2007, 2008, Ohtsuka et al. 2004).

The genus *Pseudodiaptomus* has so far accommodated 77 species (Boxshall and Halsey 2004, Walter and Boxshall 2012) and been taxonomically divided into seven species groups and four unassigned species which can be characterized mainly by sexual dimorphic features (Walter 1986a, Walter et al. 2006). In Thailand only eight species have hitherto been recorded: *Pseudodiaptomus andamanensis* Pillai, 1976, *Pseudodiaptomus aurivilli* Cleve, 1901, *Pseudodiaptomus bulbiferus* Rose, 1957, *Pseudodiaptomus clevei* A. Scott, 1909, *Pseudodiaptomus dauglishi* Sewell, 1932, *Pseudodiaptomus mertoni* Früchtl, 1923, *Pseudodiaptomus tollingerae* Sewell, 1919, *Pseudodiaptomus trihamatus* Wright, 1937 (Suvapepun 1984, Walter 1986a, Suwanrumpha 1987, Walter et al. 2002, Pinkaew 2003, Srinui 2007). The present paper deals with a detailed description of the new species of *Pseudodiaptomus* collected from Thailand with remarks on the zoogeography and ecology.

## Materials and methods

**Figure 1.**
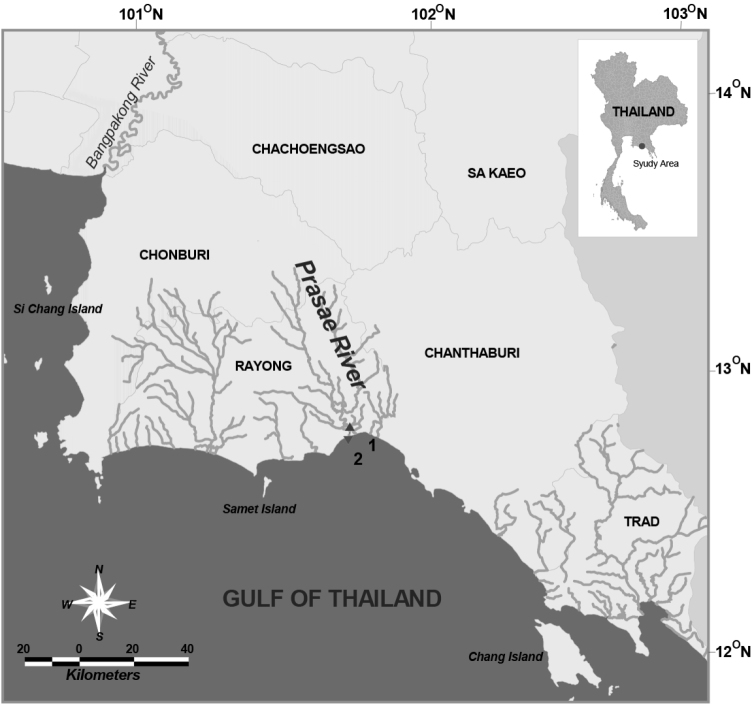
Sampling stations in Prasae River Estuary, Rayong Province.

Copepods were collected at 2 stations from the near-bottom of the Prasae River Estuary, Rayong Province, in June 2011 and August 2004, 2012 using a plankton net (0.1 mm in mesh size) and a sledge net (0.3 mm) (Fig. 1). Samples were fixed in 5% neutralized formaldehyde/seawater solution immediately after capture. Calanoid copepods were sorted out of the original samples under a stereo microscope. Copepod specimens were transferred directly from preservative to polyvinyl lactophenol and dissected with a pair of fine needles. All drawings were made with the aid of a camera lucida attached to a compound microscope (Olympus BX50). Each segment and appendage is numbered using Arabic numerals. Terminology follows Huys and Boxhall (1991). The female urosome of the new species was examined with a scanning electron microscope (Jeol-JSM-6510LV). The temperature, salinity and dissolve oxygen at the sampling site were measured at 1-m depth by using a multi-parameter probes YSI model 6600-M. The type specimens of the new species were deposited in the Institute of Marine Science, Burapha University (BIMS-Z00-0129).

## Systematics

### Order Calanoida G.O. Sars, 1903
Family Pseudodiaptomidae G.O. Sars, 1902
Genus *Pseudodiaptomus* Herrick, 1884

#### Pseudodiaptomus siamensis sp. n.

http://zoobank.org/4DE3A857-3BDE-4107-8758-F1B74325C573

http://species-id.net/wiki/Pseudodiaptomus_siamensis

Figs 2 –5

##### Material.

Prasae River Estuary, the Gulf of Thailand, station1: (12°42.66'N, 101°42.37'E; station 2: 12°41.14'N, 101°42.49'E) (Fig. 1), 23 August 2004 (6♂♂); 4 June 2011 (8♀♀, 6♂♂); 13 August 2012 (11♀♀, 1♂).

##### Types.

Holotype: 1♀ station 1, 4 June 2011, dissected and mounted on 2 glass slides (BIMS-Z00-0130), allotype: 1♂ station 1, 4 June 2011, dissected and mounted on 5 glass slides (BIMS-Z00-0131); paratypes: 4♀♀, station 1, 13 August 2012, 3 ♂♂ station 2, 23 August 2004 partly dissected and mounted on 3 glass slides (BIMS-Z00-0132).

##### Description.

**Figure 2.**
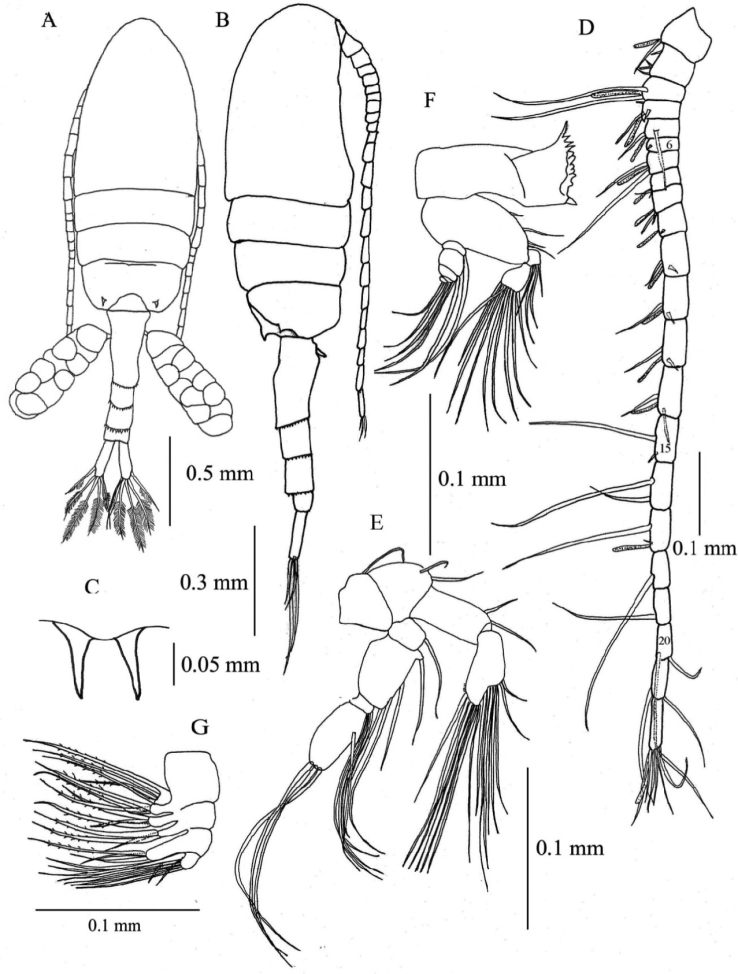
*Pseudodiaptomus siamensis*, sp. n., female (holotype). **A** habitus, dorsal view **B** habitus, lateral view **C** rostrum, ventral view **D** right antennule, arabic numerals denote segment numbers **E** right antenna **F** mandible **G** maxilla.

**Female.** Total length, 1.29–1.41 mm (mean±SD = 1.37±0.04 mm, *N*=5; holotype, 1.29 mm); prosome length, 0.75–0.82 mm (0.79±0.02 mm; holotype, 0.75 mm); prosome width, 0.31–0.34 mm (0.32±0.01 mm; holotype, 0.32 mm). Habitus (Figs 2A, B) with anterior margin of cephalosome rounded in dorsal view. Rostrum with paired filaments (Fig. 2C). Cephalosome and first pedigerous somite completely fused; fourth and fifth pedigerous somites totally fused. Prosomal ends rounded; dorso-lateral spines on fifth pedigerous somites. Urosome 4-segmented. Genital double-somite asymmetrical in dorsal view, elongate, ca. 2.54 times as long as wide; postero-dorsal and lateral margins with somewhat irregular row of spinules; in ventral view, genital area furnished with blunt, linguiform process midway, transverse rows of spinules anteriorly and paired flaps originating from genital opercula (see Fig. 5); each of paired egg-sacs consisting of 9–14 eggs, attached to lateral of genital opening (Fig. 2A). Proportional lengths of urosomites and caudal ramus 43:15: 15: 7: 20 (=100); length to width ratios 2.5, 1.3, 1.3, 0.4, and 3.6, respectively. Second and third urosomites with row of minute spinules along postero-dorsal and lateral margins. Caudal rami with hair on inner margin and symmetrical with 6 setae: seta I absent, seta II with fine setules only along inner margin; setae III-VI plumose; seta VII located dorsally.

Antennule (Fig. 2D) reaching beyond posterior end of genital double-somite, symmetrical, 22-segmented; segments 6-7 incompletely fused; segments 6, 15, 16, 18-21 each without aesthetasc (ae). Fusion pattern and setal elements as follow: 1 - 1 + ae, 2 - 3 + ae, 3 - 2 + ae, 4 - 3 + ae, 5 - 3 + ae, 6 - (1 spiniform element), 7 - 2 + ae, 8 - 2 + ae, 9 - 2 + ae, 10 - (1 spiniform element) + ae, 11-14 - 2 + ae, 15-16 - 2, 17 - 2 + ae, 18-19 - 1, 20-21 - 2, 22 - 6 + ae.

Antenna (Fig. 2E) coxa with single seta; basis with 2 setae at inner corner; endopod 2- segmented, first segment with 2 setae, second segment with 7 and 8 setae on terminal and subterminal lobes, respectively, and lateral row of fine setules; exopod 4-segmented, first segment with 1 seta, second segment with 1 proximal, 2 medial and 1 terminal setae; third segment with 3 setae; fourth segment with 1 medial and 3 terminal setae.

Mandible (Fig. 2F) with basis bearing 4 setae along inner margin; endopod 2- segmented, first segment with 4 setae, second with 9 setae; exopod 5-segmented, first to fifth segments with 1, 1, 1, 1, 2 setae, respectively. Gnathobase (coxa) with serrate dorsal seta and 3 cuspidate and 4 blunt teeth.

**Figure 3.**
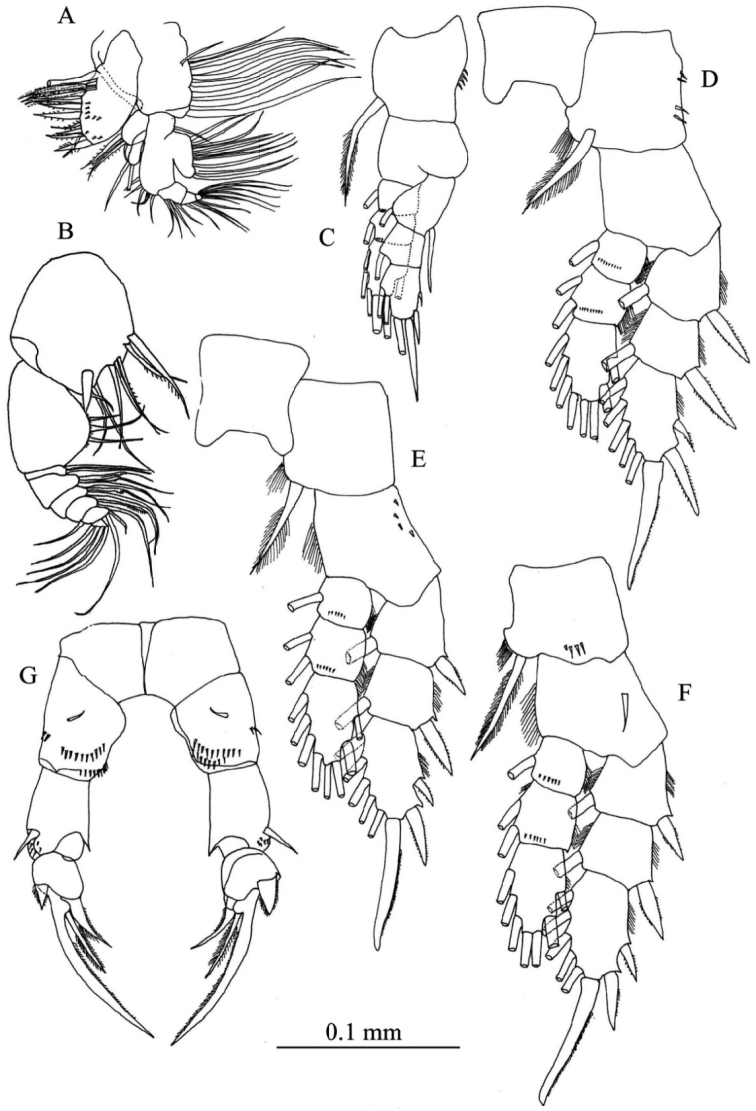
*Pseudodiaptomus siamensis*, sp. n., female (holotype). **A** maxillule **B** maxilliped **C** leg 1, posterior view **D** leg 2, posterior view **E** leg 3, anterior view **F** leg 4, posterior view **G** leg 5, posterior view.

Maxillule (Fig. 3A) with preacoxal arthrite bearing 9 strong and 6 fine setae and small spinules; coxa with 4 setae on endite and 9 setae on epipodite; basis with 4 and 5 setae on proximal and distal endites, respectively; basal exite with 1 seta; endopod 3-segmented, with 4, 4 and 6 setae from first to third segments, respectively; exopod foliaceous with 10 setae along outer margin.

Maxilla (Fig. 2G) with first and second praecoxal endites having 4 and 3 setae, respectively; first coxal endite with 3 long setae, second endite with 1 short strong and 2 long setae; basis with 1 short and 2 long setae; endopod with 9 setae.

Maxilliped (Fig. 3B) with praecoxa and coxa completely fused; endites with 0, 2, 3, 4 setae, respectively; basis with 3 setae; endopodal segment having 6 segments, first segment with 2 setae, second segment with 2 bifurcated setae and 1 seta, third and fourth segments with 1 bifurcated seta and 1 seta, fifth and sixth segments with 3 and 4 setae, respectively.

Legs 1–4 (Figs 3C–F) biramous with 3-segmented rami; coxa and basis of both rami with spinules on distal corner. Seta and spine formula as follows:

Leg 5 (Fig. 3G) uniramous and almost symmetrical; in posterior view, basis with short medial seta and spinular rows; exopod 3-segmented, first segment produced into small pointed process at inner subterminal corner, with distolateral spine and one or two rows of spinules; second segment having short and thickned disto-lateral process and medial serrate spine; third segment spiniform, tapering distally with inner spinules and proximo-medial spine.

**Male.** Total length 0.94-1.02 mm (mean±SD = 0.97±0.03, N= 4; allotype, 1.02 mm). Prosome length 0.62-0.66 mm (mean±SD = 0.64±0.01, allotype, 0.66 mm), width 0.26-0.27 mm (mean±SD = 0.26±0.005, allotype, 0.26 mm).

**Figure 4.**
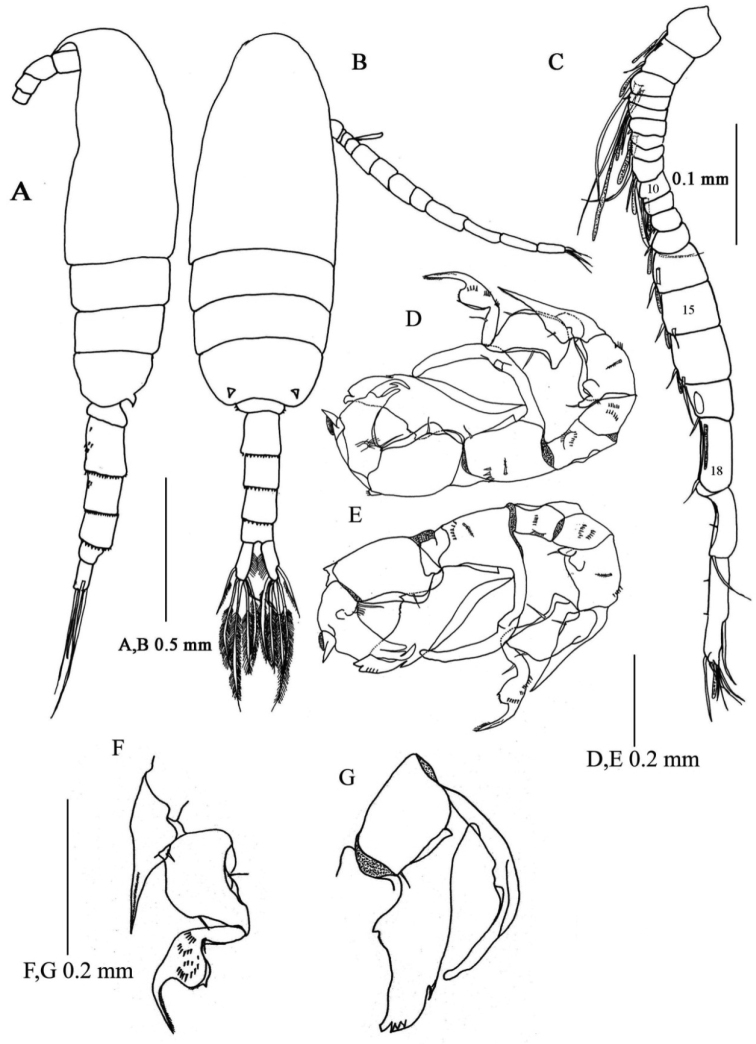
*Pseudodiaptomus siamensis*, sp. n., male (allotype). **A** habitus, lateral view **B** habitus, dorsal view **C** right antennule, arabic numerals denote segment numbers **D** leg 5, anterior view **E** leg 5, posterior view **F** anterior view of exopod of right leg 5 **G** posterior view of inner process and outer process of endopod of left leg 5.

Habitus (Figs 4A, B) similar to that of female, except for urosome. Urosome 5- segmented; proportional lengths of urosomites and caudal ramus 13: 25: 21: 17: 11:13 (=100); length to width ratios 0.5, 1.1, 1.2, 1, 0.6 and 1.7. Genital somite nearly symmetrical with one or two rows of spinules ventrally. Urosomites 2–4 with spinular row along posterior margin. Caudal rami symmetrical, with six setae as in female.

Right antennule (Fig. 4C) geniculate and indistinctly 20-segmented; setal formula as follows: 1 -1 + ae, 2 - 2 + ae, 3 - 2 + ae, 4 - 1, 5 -1 + ae, 6 - (1 spiniform element), 7 - 1 + ae, 8 - (1 spiniform element), 9 - 2 + ae, 10 - (1 spiniform element), 11 - 1 + ae, 12 - (1 spiniform element) + ae, 13 -1 + ae, 14-16 - 2 + ae, 17-18 - 1 + (1 process), 19 - 2 + (1 process), 20 - 9 + ae.

**Figure 5.**
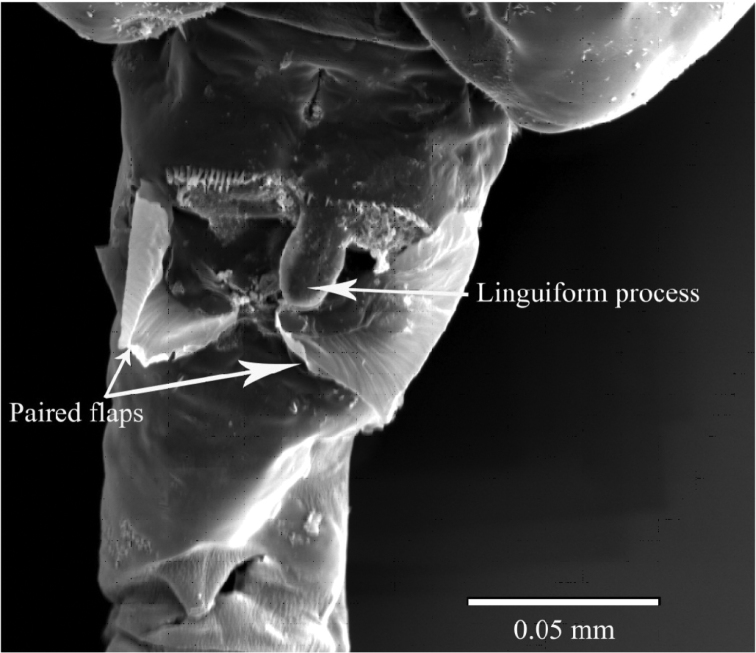
SEM micrograph of ventral side of genital double-somite of female *Pseudodiaptomus siamensis*, showing blunt process anterior to genital area.

Leg 5 (Figs 4D, E, F, G) highly asymmetrical and biramous; intercoxal sclerite and both coxae fused; coxa with fine spinular rows on anterior surface. Right leg (Figs 4D, E) with basis having outer spinular row; endopod rudimentary, represented by knob-like process with fine setule at tip; exopod (Fig. 3F) 3-segmented, first segment protruded into outer process reaching middle of third segment, proximal process with 1 spine and spinular row; second segment expanded midway, each side with spine; third segment curved inward with 3 rows of spinules on anterior surface and middle swelling, distal to which tapering distally. Left leg (Figs 4D, E) with elongated basis having triangular process at midlength; endopod (Fig. 4G) highly developed, bifurcated, inner medial process smoothly curved outward reaching distal tip of second exopod, outer process thickened, foliaceous with 1 subterminal and 4 thin terminal protrusions; exopod 2-segmented, first segment as long as basis, irregularly sinuated along inner margin; second segment triangular with hirsute process proximally and stout serrated protrusion at medio-lateral margin, with 3 processes of unequal length terminally.

##### Remarks.

The present new species can be definitely assigned to the *lobus* species group sensu Walter (1986a, b, 1987) and Walter et al. (2006) in having a combination of the following features: (1) paired egg-sacs; (2) a fusion between the cephalosome and first pedigerous somite; (3) the presence of a large endopod of male left leg 5; (4) the presence of a rudimentary endopod of male right leg 5. Its estuarine habitat in the West Pacific also supports this assignment (see Walter et al. 2002). In this species group two subgroups, *forbesi*-subgroup and *poppei*-subgroup, are distinguished and can be readily differentiated by the terminal shape of the endopod of male left leg 5: bifid (*poppei*-subgroup) or not (*forbesi*-subgroup) (Walter 1986a). The new species with a bifid tip of the endopod clearly belongs to the *poppei*-subgroup. The following four species have so far been accommodated: *Pseudodiaptomus poppei* Stingelin, 1900, *Pseudodiaptomus smithi* Wright, 1928, *Pseudodiaptomus tollingerae* Sewell, 1919, and *Pseudodiaptomus siamensis* sp. n.

In the *poppei*-subgroup the new species is most closely related to *Pseudodiaptomus tollingerae* from the Indian waters (Pillai 1976, Reddy and Radhakrishna 1982) in sharing the following features: (1) the genital double-somite of female is relatively elongate; (2) the right endopod of male leg 5 is rudimentary; (3) the terminal exopodal segment of male right leg 5 is swollen midway; (4) the shape of the left endopod of male leg 5 is similar between the two species; (5) the terminal exopodal segment of male left leg 5 bears 3 stout processes terminally. However, the new species can be easily distinguished from *Pseudodiaptomus tollingerae* in: (1) the presence of a ventral linguiform process on the genital double-somite in the female (absent in *Pseudodiaptomus tollingerae*); (2) the second exopodal segment of male right leg not so swollen proximally (swollen); (3) the proximal process of the left endopod of male leg 5 smoothly curved outward (abruptly curved at mid-length); (4) the distal process of the left endopod of male leg 5 tapering distally (expanded terminally and divided at tip). In addition, the female of the new species is unique in having a small, inner terminal process on the first exopodal segment of leg 5, which is much more conspicuous in the three known species of the subgroup.

##### Etymology.

The species was named after the type locality “Siam” (an old name of Thailand).

## Disscussion

### Zoogeography

Walter et al. (2002) recognized three types of the distributional patterns in the *lobus* species group: Type I = wide distribution of the Indo-West Pacific; Type II = confined distribution mainly or restrictedly in the Indian Ocean; Type III = confined distribution mainly or restrictedly in the West Pacific. In the *poppei*-subgroup of the species group, *Pseudodiaptomus tollingerae* is assigned to Type I, while *Pseudodiaptomus poppei* and *Pseudodiaptomus smithi* to Type III (Pillai 1976, Walter 1986a, Walter et al. 2002). *Pseudodiaptomus siamensis* has so far been recorded only from the type locality or the Gulf of Thailand, and tentatively belongs to Type III. It seems that the *poppei*- subgroup is highly restricted to estuarine waters of the Indo-Malayan realm.

As mentioned above, *Pseudodiaptomus siamensis* composes a sister group with *Pseudodiaptomus tollingerae*. *Pseudodiaptomus poppei* from Celebes (Walter 1986a, b) and *Pseudodiaptomus smithi* from the Phillipines (Walter 1986b) share synapomorphic characters such as an elongated terminal segment of male right leg 5. Therefore the distributional pattern of these two pairs in the *poppei*-subgroup implies a speciation around the Huxley’s line. A recent molecular analysis of the Indo-West Pacific populations of the giant freshwater prawn *Macrobrachium rosenbergii* Murphy & Austin (2002), using 16S ribosomal RNA mitochondrial DNA, clearly recognized two clades, each of which is located on either eastern or western side of Huxley’s line (Bruyn et al. 2004). Actually these two clades are suggested to represent two distinct species based on great sequence divergences (6.2 % in maximum) (Bruyn et al. 2004). Although exact vicarious events around Huxley’s line are still unknown, the scenario might be applied to the speciation of the *poppei*-subgroup of *Pseudodiaptomus* occurring in the brackish waters. The important point is that prawn also needs estuarine environments for reproduction (Bruyn et al. 2004).

### Ecology

The habitat of the present new species, the Prasae Estuary was euryhaline, where the salinity widely ranged between 0.7 and 23.3 during the present investigation. Dominant copepods that co-occurred with the new species seasonally differed with salinity: *Acartia plumosa* Scott, 1894, *Bestiolina similis* Sewell, 1914, *Parvocalanus crassirostris* Dahl, 1894, *Pseudodiaptomus annandalei* Sewell, 1919, and *Oithona simplex* Farran, 1913, were abundant in the wet season (May–October), while *Bestiolina similis*, *Parvocalanus crassirostris*, *Oithona simplex*, and *Oithona dissimilis* Lindberg, 1940 in the dry season (November-April) (Srinui 2007). In the estuary other environmental factors such as water temperature and dissolved oxygen were nearly constant throughout the investigation, 28.1 to 29.5 °C and 4.3 to 5.3 mg/L, respectively.

Although our collections of planktonic copepods were intermittently carried out, some information of the breeding of the new species was obtained. The ovigerous and/or spermatopore-bearing females of the new species were found during the wet season (June to October). In addition, the density of immature females reached 139 individuals per cubic meter in August 2004, suggesting it was an active breeding season.

### Key to species of the *poppei*-subgroup

Seventy-eight species of *Pseudodiaptomus*, including the new species *Pseudodiaptomus siamensis*, have been recorded from the world (Walter 1986a, 1987, Walter et al. 2002, Walter et al. 2006, present study). Walter (1984, 1986a, 1987) has also recognized seven species groups in *Pseudodiaptomus* based mainly on sexual dimorphic features. The *lobus* species group, to which the present new species belongs, has so far accommodated two subgroups and 15 species. The new species is classified into the *poppei*-subgroup with 4 species. A key to 4 species of the subgroup is provided here.

**Female**

**Table:** 

1	First urosomite symmetrical without blunt linguiform process on mid-ventral	2
–	First urosomite asymmetrical with blunt linguiform process on mid-ventral	*Pseudodiaptomus siamensis*
2	First urosomite with pair of anterodorsal spines and posterodorsal cluster of 3 spinules	*Pseudodiaptomus smithi*
–	First urosomite without pair of anterodorsal spines and posterodorsal cluster of 3 spinules	3
3	Prosomal ends with one pair of processes dorsally	*Pseudodiaptomus tollingerae*
–	Prosomal ends with two pairs of processes dorsally	*Pseudodiaptomus poppei*

**Male**

**Table:** 

1	Fifth pair of legs without left endopodal segment	*Pseudodiaptomus poppei*
–	Fifth pair of legs with left endopodal segment	2
2	First exopodal segment of fifth right legs with recurved process at distolateral corner	*Pseudodiaptomus smithi*
–	First exopodal segment of fifth right legs with straight process at distolateral corner	3
3	Endopod of fifth left leg with outer process tapering distally	*Pseudodiaptomus siamensis*
–	Endopod of fifth left leg with outer process concave at the tip	*Pseudodiaptomus tollingerae*

## Supplementary Material

XML Treatment for Pseudodiaptomus siamensis
